# Outcomes in adulthood after neurosurgical treatment of brain tumors in the first 3 years of life: long-term follow-up of a single consecutive institutional series of 97 patients

**DOI:** 10.1007/s00381-020-04859-1

**Published:** 2020-08-19

**Authors:** Tryggve Lundar, Bernt Johan Due-Tønnessen, Radek Frič, Petter Brandal, Einar Stensvold, Paulina Due-Tønnessen

**Affiliations:** 1 Department of Neurosurgery, Oslo University Hospital, Postboks 4950 Nydalen, 0424 Oslo, Norway; 2 Faculty of Medicine, University of Oslo, Oslo, Norway; 3 Department of Oncology, Oslo University Hospital, Oslo, Norway; 4 Department of Paediatrics, Oslo University Hospital, Oslo, Norway; 5 Department of Radiology, Oslo University Hospital, Oslo, Norway

**Keywords:** Infantile brain tumors, Adult outcome, Pediatric neurosurgery

## Abstract

**Background:**

Long-term outcome for children who underwent surgery for brain tumors in the first 3 years of life is not well-known.

**Methods:**

We performed a retrospective study on surgical morbidity, mortality rate, academic achievement, and work participation in children below 3 years of age who underwent primary tumor resection for a brain tumor in the period from 1973 to 1998. Gross motor function and activities of daily life were scored according to the Barthel Index. Long-term survivors were defined as with a survival from primary diagnosis of 20 years or more.

**Findings:**

Ninety-seven consecutive children were included. No patient was lost to follow-up. Gross total resection was achieved in 67 children during the primary procedure, 25 had subtotal resections, and 5 had only partial resection. The 20-year survival figures for the 46 children with high-grade tumors was 33%, and the corresponding figures for 51 patients treated for low-grade tumors was 82%. Five of the 57 20-year-survivors died 21, 29, 30, 30, and 41 years, respectively, following primary surgery. Fifty of the 52 long-term survivors had a Barthel Index (BI) of 100, while the remaining two had a BI of 40. Twelve patients were long-term survivors after treatment for HG tumors (26%), while 40 of the 51 patients treated for LG tumors (78%) were alive. Thirty-two of the 52 long-term survivors were in full-time work and 29 of them after treatment for LG tumors. Another 10 were in part-time work, while the last 10 individuals had no working capacity.

**Conclusion:**

Survival is better for patients with low-grade tumors compared with those with high-grade tumors. The functional level of long-term survivors is affected by adjuvant therapy and radiotherapy in particular. Neurosurgical intervention in itself is safe and plausible for pediatric brain tumor patients below 3 years of age. However, there should be a focus on potential late affects, and survivors should be followed by knowledgeable clinical staff for the neoplastic disease as well as for potential side effects. In this consecutive series, a 33% 20-year survival for children treated for HG tumors and 82% for patients with LG tumors was observed. The patients with LG tumors who had been treated with surgical resection without any adjuvant therapy showed a good clinical outcome as adults, and two-thirds of them were in full-time work.

## Introduction

Brain tumors occurring in early life years are considered to be a disastrous event for the child and its family [1, 3, 9, 15]. High-grade (HG) tumors are often very aggressive, and few small children with posterior fossa medulloblastoma became long-term survivors, until the introduction of postoperative radiotherapy at our institution in 1974, after which half of the children with this disease survived for 20 years [13, 14]. The deleterious effects of postoperative radiotherapy were recognized during the late 1980s, and treatment without up-front radiotherapy became standard for children below 3 years of age. Major neurosurgical tumor resections also represent a potential threat to infants with brain tumors, along with the neoplastic disease itself. In addition, many of these children also require surgical treatment for secondary hydrocephalus (HC), and adjuvant chemotherapy has major side effects.

Although deleterious side effects of different treatment strategies have been debated for several decades, outcome has mostly been discussed in terms of survival rates, while long-term side effects for small children treated for brain tumors have not been extensively reported [5, 11]. In a previous study on 30 consecutive children treated in their first 6 months of life, we found that some of them did better than expected, but follow-up was short for the majority of the patients [5]. Thirteen of these 30 children treated before 1998 are included in the present study. Our next study included 34 patients treated in their first year of life before 1998. This would imply that all survivors were true 20-year survivors and have become adults. The long-term result could therefore also include working capacity and was published in 2019 [8]. In the present study, our aim was to analyze the functional level for all brain tumor survivors treated before the age of 3 years and with more than 20 years of follow-up. The previous study on the 34 youngest individuals was therefore expanded to 97 consecutive children including also those treated in the second and third year of life.

## Methods

We retrospectively analyzed a nonselected, consecutive cohort consisting of children under the age of 3 years who underwent primary resection of a brain tumor between 1973 and 1998 at Department of Neurosurgery, Oslo University Hospital, Oslo, Norway. We defined long-term survivors as patients who had at least 20 years follow-up, and the clinical situation of all survivors at the end of 2018 is described. Cases were identified and collected based on surgical protocols from the study period. The case record data included sex, age at the time of primary tumor resection, and data on repeated operations and management of secondary hydrocephalus. To assess functional status, Barthel Index (BI) score was used [10]. This is a well-established and validated scale using ten variables to measure performance in basic activities of daily living (ADL) primarily related to personal care and mobility. Scores range from 0 to 100; a higher score denotes greater independence. Educational outcome was simplified and categorized into normal versus special schooling, and employment attendance into open, sheltered, or no work.

Grade of resection was based on the surgeons preoperative evaluation and, whenever performed, postoperative radiological examination (CT, MRI). Gross total resection (GTR) means full tumor resection judged by the surgeon without residual tumor on postoperative CT/MRI, subtotal resection (STR) means at least 90% resection of tumor, and partial resection means non-GTR/STR.

## Results

We identified 97 patients, 52 boys and 45 girls (ratio 1.15:1), with a median age of 1.4 (range 0–2.9) years. All children younger than 3 years of age in which a primary brain tumor resection had been performed were included. These patients represented 14% of all central nervous system tumors operated between 1973 and 1998. Thirty-four children underwent primary surgery in their first year of life, while 29 and 34 patients were in their second and third year, respectively.

The histological examination was performed by an experienced neuropathologist and revealed a high-grade tumor (WHO grade III or IV) in 46 patients (Table 1). The most common malignant tumor was primitive neuroectodermal tumor (PNET), with 19 cases located to the posterior fossa (medulloblastoma) and 6 supratentorial cases.

**Table 1:** Histological findings from 97 consecutive children aged 0–2 years operated (1973–1998) for brain tumors with at least 20 years follow-up

Histology	High-grade	Low-grade
Astrocytoma	7	30
Plexus tumor	4	12
Teratoma	1	2
Ganglioglioma		2
Oligodendroglioma		2
DNET		1
PXA		1
Craniopharyngioma		1
Ependymoma	7	
PNET	25	
ATRT	1	
Sarcoma	1	
Total	46	51

ATRT, atypical teratoid rhabdoid tumor; DNET, dysembryoplastic neuroepithelial tumor; PNET, primitive neuroectodermal tumor; PXA, pleomorphic xanthoastrocytoma

The most common low-grade tumor was astrocytoma (Table 1) with 30 cases out of 51. Sixteen of these were located to the posterior fossa and five of them in the brain stem. Among the fourteen supratentorial low-grade astrocytomas, 8 were suprasellar tumors affecting chiasma-hypothalamic functions; five were pilocytic grade I tumors and 3 grade II tumors.

### Tumor location and clinical presentation

The tumor was localized in the supratentorial compartment in 50.5% and in the posterior fossa in 49.5% of patients. Signs and symptoms of increased intracranial pressure (vomiting, rapid head growth, sun set sign) were the leading clinical presentation. Only 3 children presented with seizures and two patients with tetraplegia and respiratory arrest.

### Surgical resection

Gross total resection (GTR) was achieved in 67 patients and STR in 25 cases, and the remaining 5 patients underwent a partial resection. Twelve children died in the early postoperative period (1 month) after primary surgery. In total, 18 children received shunts due to secondary hydrocephalus, many of them requiring later shunt revisions.

### Repeated resection

Four patients with HG tumors underwent repeated tumor-resective surgery (Table 2). This was the case in two children with choroid plexus carcinoma who demonstrated residual tumor on postoperative MRI and in two children with recurrent posterior fossa ependymoma approximately 1 year following primary surgery.

**Table 2:** Clinical details from high-grade tumor patients with more than 20-year survival

Patient no.	Sex Age	Year	Histology	Location	Resection grade	Alive	Present age (years)	Follow-up (years)	Work status	Adjuvant treatment	Morbidity
1	M2	1975	PNET	PF	GTR	Yes	45	43	FTW-No	RT	Major stroke 2012
2	F0	1975	PNET	PF	GTR	No	Dead	41	–	RT	Meningiomas, BCC, HC
3	M0	1978	PNET	PF	GTR	No	Dead	30	–	RT	HC, heart valve affection
4	F0	1982	GBM	Right frontal	GTR	No	Dead	29	–	RT	Multiple meningiomas
5	M1	1982	PNET	PF	GTR	Yes	37	36	PTW	RT + Ch	–
6	F0	1983	Ependymoma	PF	GTR	Yes	36	35	No	RT	Repeated strokes
7	M2	1983	PNET	PF	GTR	Yes	38	36	PTW-No	RT + Ch	Meningiomas, epilepsy, heart valve affection
8	F2	1985	PNET	PF	GTR	Yes	36	34	PTW	RT	Meningiomas, rectal ca, outer ear ca, epilepsy
9	M1	1987	Plexus ca	IIIv	STR	Yes	33	32	FTW	RS	HC, ETV
10	F2	1987	PNET	PF	STR	Yes	34	32	PTW	RT + Ch	Meningioma, HC, BCC
11	F1	1988	PNET	IIIv	STR	Yes	32	31	PTW	RT + Ch	HC, epilepsy
12	M0	1988	Plexus ca	lv	STR	Yes	30	30	No	RS	HC, epilepsy
13	F2	1990	Sarcom	Right frontal	GTR	Yes	29	28	FTW	No	–
14	M1	1993	PNET	PF	GTR	Yes	26	25	FTW	Ch, RS	HC
15	F2	1998	Ependymoma	PF	GTR	Yes	24	21	PTW	No	HC, shunt failure, VPS

IIIv, third ventricle; BCC, basal cell carcinoma; Ch, chemotherapy; FTW, full-time work; HC, hydrocephalus; lv, lateral ventricle; PF, posterior fossa; PTW, part-time work; RS, repeated surgery; RT, radiotherapy; VPS, ventriculo-peritoneal shunt

**Fig. 1:**
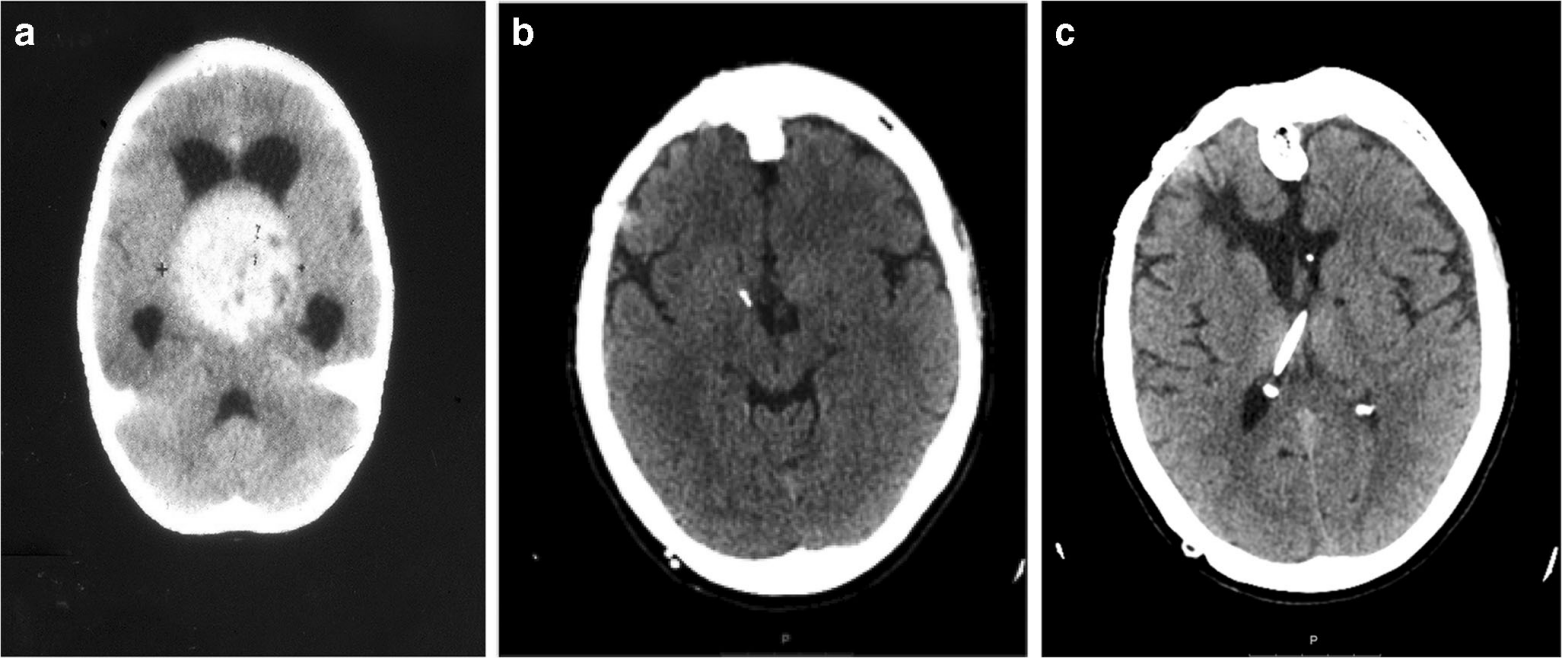
A 2-year-old girl presenting in 1981 with severe headache, ataxia, and visual impairment. CT demonstrated a large 3rd ventricular tumor (**a**). After STR of low-grade astrocytoma and subsequent hormonal substitution, she has also been shunt-dependent for HC and needed repeated resections in 1994 and 2009 due to progression of residual tumor. In spite of left-sided amaurosis, she is today at the age of 40 years in part-time work with a CT indicating tumor control (2019, **b**, **c**)

Ten of the 51 patients with LG tumor underwent more than one surgical resection (Table 3). In one of them, this was caused by multiple tumor locations (bilateral plexuspapillomas). In 9 patients, a second resection was performed due to recurrence or progression of residual tumor, and in three of these, a third resection was also performed (intra-axial lipoma in medulla oblongata, supratentorial DNET, 3rd ventricle astrocytoma (Fig. 1).

**Table 3:** Clinical details for low-grade tumor patients with more than 20-year survival

Pat.nr Sex Age	Year	Histology localization	Res grade	Outcome Barthel Index	F-up (years)	Present age (years)	Work?	Adj. Treatment	Comments
1 F0	1975	FPA	GTR	100	43	43	FTW		
2 F1	1975	FPplexpap	GTR	100	43	44	FTW		
3 M2	1976	FPA	GTR	100	42	45	FTW		Spinal trauma 2001
4 F2	1977	FPA	STR	–	30	Dead	–	HC	Shunt failure
5 F1	1978	PlexpapLV	GTR	100	40	41	FTW		
6 M1	1979	Astro. suse	STR	100	39	41	No		Psychosis
7 M0	1980	Astro. suse	STR	100	38	39	FTW		
8 F2	1980	FPA	GTR	100	38	41	No		PNES
9 F0	1980	DIGsupra	GTR	100	38	39	PTW	HC	
10 M1	1981	Oligo. sup	STR	100	37	38	PTW- No	RS	Epi-surg2017
11 F2	1981	Astro-IIIv	STR	100	37	39	PTW	RS,HC	RS-94 + 09
12 M0	1981	Plexpap IIIv	GTR	100	37	37	FTW		
13 M1	1982	FPA	GTR	100	36	38	FTW		
14 F0	1982	Plexpap LV	GTR	100	36	37	FTW		
15 M2	1983	Astro. suse	STR	100	35	37	FTW	RS,RT	RS-92, gamma
16 M2	1985	Astro. sup	GTR	100	33	35	FTW		
17 F2	1986	Plexpap. LV	GTR	100	31	33	FTW		
18 M2	1987	Astro.sup	GTR	100	31	33	FTW		
19 F2	1987	Astro. sup	GTR	100	31	33	FTW		
20 M0	1987	Plexpap. LV	GTR	100	31	31	FTW		
21 M1	1988	Astro. IIIv	STR	–	19	Dead	–	RS	HC
22 F2	1989	Astro. sup	STR	–	21	Dead	–	Ch, RT	
23 M0	1989	Astro. sup	GTR	100	29	29	FTW		
24 F1	1990	Plexpap. LV	GTR	40	28	29	No		Bilat tumors
25 M1	1990	Astro. sup	GTR	100	28	29	FTW		
26 F1	1990	DNET. sup	GTR	100	28	29	FTW	RS	RS-94, -97
27 M2	1990	FPA	GTR	100	28	31	FTW		
28 F0	1990	FPlipoma	STR	40	28	28	No*	RS	RS-07, 09; HC
29 F2	1990	Astro. sup	STR	100	28	30	PTW	RS	RS-92, epilepsy
30 M1	1990	Astro. suse	STR	100	28	29	FTW		
31 M0	1991	FPA	GTR	100	28	28	FTW		
32 M0	1991	Plexpap. IIIv	GTR	100	27	27	FTW		
33 F1	1991	FPA	GTR	100	27	28	FTW	RS	RS-01
34 M2	1992	FPA medobl	STR	–	17	Dead	–	RS	RS-93
35 M1	1992	FPA	GTR	100	26	27	FTW	RS	RS-95
36 M1	1992	Oligo. sup	GTR	100	26	27	FTW		
37 M2	1993	Astro. sup	STR	100	25	27	PTW; no		
38 F2	1994	FPA	GTR	100	24	27	FTW		
39 F1	1995	FPA	GTR	100	24	26	FTW		
40 M0	1995	PXA. sup	GTR	100	23	24	PTW		
41 F1	1995	FPggl	GTR	100	23	25	PTW	HC	Shunt dependent
42 M0	1997	Plexpap LV	GTR	100	21	21	FTW		
43 M2	1998	Teratom. sup	STR	100	20	23	FTW		
44 M0	1998	Plexpap LV	GTR	100	20	20	FTW		

PF, posterior fossa; GTR, gross total resection; Sup, supratentorial; STR, subtotal resection; Suse, suprasellar; FTW, full-time-work; LV, lateral ventricle; PTW, part-time-work; IIIv, third ventricle; RS, repeated surgery; Medobl, medulla oblongata; HC, hydrocephalus; A, Astro, astrocytoma; No*, no work, but student; Plexpap, plexus papilloma; RT, radiotherapy; Ggl, ganglioglioma; DIG, diffuse infiltrating ggl; Oligo, oligodendroglioma; PXA, pleomorphic xanthosatrocytoma

### Survival

In total, 52 of the 97 children are still alive at latest follow-up, and the observed 20-year survival was 59%. For patients with LG tumors, 20-year survival was 82%, and for HG tumors, it was 33% (Table 4). Forty of the 51 patients (78%) with LG tumors were alive after a median follow-up of 32 years (range 20–45 years). Looking at tumor location, 29 of the 49 (59%) infants with supratentorial tumors were alive at latest follow-up, while the observed 20-year survival was 63%. Only nine of the 48 (19%) patients with posterior fossa tumors were alive, but the observed 20-year survival rate was 25%.

**Table 4:** Observed survival rates in 97 consecutive children (age 0–2 years) operated for brain tumor (1973–1998) with at least 20 years follow-up

	Number	1 month	1 year	5 years	10 years	15 years	20 years	Alive	Late deaths
0–2	97	0,90 87/97	0,70 68	0,61 59	0,61 59	0,61 59	0,59 57	0,56 52	7 patients
High-grade	46	0,87 40/46	0,50 23	0,33 15	0,33 15	0,33 15	0,33 15	0,26 12	29, 30, 41 years
Low-grade	51	0,92 47/51	0,88 45	0,86 44	0,86 44	0,86 44	0,82 42	0,78 40	17, 19, 21, 30 years

### Adjuvant therapy

Only children with HG tumors received adjuvant therapy. Of the 46 patients, 7 were given postoperative radiotherapy, and some also received chemotherapy (Table 2). During the last 10 years of the study period (after 1987), up-front radiotherapy as part of primary treatment was abandoned in this age group due to the accumulating data on its detrimental effects.

### Activities of daily life

All except two long-term-survivors had a good gross motor function with a Barthel Index score of 100. The two remaining patients had a Barthel Index score of 40.

### School, education, and work

All 15 children with HG tumors group surviving for 5 years or more reached school age and 20-year survival. Many of them needed assistance and special schooling; however, all but three of them could perform simple practical sheltered work as teenagers. The latter three deteriorated clinically and died 29, 30, and 41 years after primary diagnosis, and all three were given radiotherapy in their first year of life (Table 2). The remaining 12 long-term survivors from the HG group were aged 21–45 years with follow-up of 21–43 years. Three of them were in full-time work and five in part-time work. Five had no working capacity at the time of analysis, but two had worked for years until they experienced severe long-term side effects from treatment (Table 2).

All 44 children with LG tumors who survived for 5 years (86%) reached school age and 15-year survival. Two of them experienced neoplastic progression and died 17 and 19 years from primary diagnosis (Table 3), leading to a 20-year survival of 82% for LG tumors. There were two late deaths after 21 and 30 years (Table 4). The remaining 40 long-term survivors in the LG group were aged 20–45 years at time of analysis, with follow-up of 20–43 years. Twenty-nine of the 40 patients (72%) were in full-time work and five patients (12%) in part-time work, while the remaining six patients (15%) had no working capacity (Table 3).

## Discussion

This report presents complete follow-up data of all children under 3 years of age who underwent operative treatment for brain tumors during a period of 25 years at our institution. As the prognosis for these patients has traditionally been considered dismal, particularly for patients with HG tumors, the study period was chosen to assess long-term survival figures and to highlight data on educational outcome and working ability among long-term survivors.

As expected, the survival figures for patients treated for high-grade tumors were poorer than the corresponding figures for patients with low-grade tumors. Nonetheless, 33% of patients with HG tumors became long-term survivors. It is very important, however, to highlight the clinical function of these 15 patients. Several of them were affected by severe late effects including second neoplasms, cerebrovascular disease, and coronary valvular disease. Three died at the age of 29, 30, and 41 years, most probably due to late effects of radiotherapy given in their first year of life. None of the seven patients still alive after treatment including radiotherapy were in full-time work, whereas three of the five survivors treated without radiotherapy are in full-time work. This observation underscores the potentially detrimental side effects from radiotherapy [3]. Hopefully, newer antineoplastic management strategies without such a high risk of serious late effects will be developed, and the advent of new radiotherapy modalities such as proton therapy should be taken advantage of whenever possible. Nonetheless, a close follow-up of these patients keeping focus on potential late effects of treatment will be crucial also in the future and should be prioritized higher than today.

The results for the LG tumor group is as expected better when it comes to survival figures, and it is rewarding to see that also long-term clinical outcome is better than for patients with HG tumors. Some patients (14%) died in the first 5 years, but thereafter survival was stable. No patient received adjuvant oncological treatment, whereas ten patients (22%) went through new tumor-resective surgery. Of the four late deaths, three were caused by neoplastic progression of midline astrocytomas 17, 19, and 21 years after primary surgery. If treated today, these patients might have been candidates for modern radiotherapy at tumor progression. The latter late death was caused by an acute shunt failure which is exceedingly rare, but the risk of such failure is impossible to fully eliminate [12]. The fact that 72% of long-term survivors in this LG tumor group had full-time working capacity is important and very interesting. The message is that also pediatric brain tumors in children below 3 years of age can be safely treated and become well-functioning survivors in adulthood. The results confirm that many children with specific low-grade tumors can be managed with resective surgery alone as pointed out previously [2, 4–8].

## Conclusion

The results from this study illustrate some major points important to communicate when counseling parents of small children with brain tumors. First and as expected, survival is better for patients with low-grade tumors compared with those with high-grade tumors. There is, especially for patients with high-grade tumors, an unmet need for more effective antineoplastic measures. Also, the functional level of long-term survivors is affected by adjuvant therapy and radiotherapy in particular. If possible, radiotherapy should be avoided, and if needed, it should be as gentle as possible taking advantage of new technology. Neurosurgical intervention in itself is safe and plausible for pediatric brain tumor patients below 3 years of age, and many survivors become well-functioning adults. However, there should be a focus on potential late effects, and survivors should be followed by knowledgeable clinical staff for the neoplastic disease as well as for potential side effects. We hypothesize that the latter could be followed in dedicated late effect clinics.
